# Minimal Detectable Change in Resting Blood Pressure and Cardiorespiratory Fitness: A Secondary Analysis of a Study on School-Based High-Intensity Interval Training Intervention

**DOI:** 10.3390/jcm12196146

**Published:** 2023-09-23

**Authors:** Jarosław Domaradzki

**Affiliations:** Department of Biostructure, Wroclaw University of Health and Sport Sciences, 51-612 Wroclaw, Poland; jaroslaw.domaradzki@awf.wroc.pl

**Keywords:** school-based high-intensity interval training, minimal detectable change, body composition, resting blood pressure, cardiorespiratory fitness

## Abstract

High-intensity interval training (HIIT) effects on resting blood pressure (BP) and cardiorespiratory fitness (CRF) have already been studied. Furthermore, the responses of responders and non-responders to HIIT in terms of these physiological outcomes have also been examined. However, the minimal detectable change (MDC) in BP and CRF has not been addressed yet. Therefore, the current study aimed to compare the MDC_90_ of BP (systolic and diastolic) and CRF (fitness index (FI) results) in the context of a school-based HIIT program for adolescents. Participants were adolescents, with an average age of 16.16 years (n = 141; 36.6% males). A preplanned secondary analysis was conducted using pre–post data from the control group to estimate MDC_90_. The MDC_90_ of SBP, DBP, and FI were 7.82 mm HG, 12.45 mm HG, and 5.39 points, respectively. However, taking into account the relative values of these changes, MDC_90_ required a greater change in DBP (17.27%) than FI (12.15%) and SBP (6.68%). Any training-induced physiological changes in the average values of the outcomes did not exceed MDC_90_. However, a comparison of the participants who exceeded and did not exceed MDC_90_ showed statistically significant differences. These findings reveal the huge variability in and insensitivity to the intervention effect for all measurements. This is likely because of the large subgroup of participants with low sensitivity to the physiological stimulus. As such, there is a considerable need to create individually tailored intervention programs.

## 1. Introduction

The common global problems related to cardiovascular health are elevated blood pressure (BP) and low cardiorespiratory fitness (CRF) [1]. What is more, cardiovascular diseases associated with low physical efficiency and obesity appear increasingly in childhood and adolescence [2]. One of the main reasons is a lack of physical activity. Generally, young people (13–17 years old) need 60 min of moderate-to-high-intensity physical activity daily [3]. This minimum dose of exercise is a guarantee of health benefits. However, the prevalence of inactivity is increasing. It is estimated that about 80% of young people do not perform the minimum physical activity level recommended by the World Health Organization (WHO) [4]. What is more, even physically active people reduce their activity by about 10% annually during adolescence [5]. Thus, research is looking into ways to ensure greater physical activity regardless of young people’s leisure time. The solution seems to be high-intensity interval training (HIIT) introduced into typical physical education (PE) lessons [3].

The HIIT method uses a short intervention time (up to a few minutes) with vigorous-intensity exercise. Evidence has confirmed that HIIT improves adolescents’ maximum oxygen uptake and reduces WHR, BMI, and body fat percentage [6,7]. However, the aforementioned studies were conducted mainly in clinical conditions, instead of natural conditions, e.g., during school PE classes. Interest in the health effects for adolescents resulting from the inclusion of HIIT in PE lessons is constantly growing [8,9]. The pros, besides the health benefits, are a short time of effort (a few minutes can be easily implemented into a typical 45 min lesson), a circuit training method (which is easy to arrange), and simple exercises based on jumps, push-ups, squats, etc., which are easy to execute and there is no need to learn extra techniques. However, a separate problem is the assessment of the real effects of HIIT.

Assessing participants’ progress (e.g., clinical, sport, or PE) is an integral part of any intervention practice. Recognizing meaningful effects is key to evaluating a training program’s effectiveness or predicting positive changes in the target variables. Many studies, particularly clinical studies, have attempted to reveal threshold change values, which may assist clinical decision making regarding a patient’s change status [10]. The results have allowed for the conclusion that the chances of health improvement in patients are predicted by certain assessment values and a postmeasure chance of improvement. The authors also noted that these values are dependent on the premeasure chance of improvement. The claim that a change is meaningful is based on the remarks that the previous and postintervention assessment values for the outcome of interest meet an established threshold value. To date, many threshold change values have been published for many outcomes, although mostly clinical ones, including patient-rated psychometric instruments and outcomes measured in patients with osteoarthritis, heart disease, brain stroke, or spinal cord injury [11,12,13]. Unfortunately, there are no such data for interventions directed at typical healthy individuals, particularly those in the developmental period of life.

Previous research on meaningful changes has focused primarily on the minimal (clinical) important difference (M(C)ID) and minimal detectable change (MDC) [14]. M(C)ID represents the smallest amount of change in an outcome that might be considered important by the participant or judge, while MDC is the minimum amount of change in a participant’s score that ensures that the change is not the result of measurement error [15]. Such analyses are useful in interpreting results, both in individuals and in groups of patients participating in controlled trials and in the planning of new trials. Regardless of the advantages and disadvantages of both strategies, which can be read in more detail in dedicated articles [16,17], MDC was used in this work. A change in outcome can be interpreted as a true difference between the baseline measurement and a second measurement only if the observed change between the two measurements is larger than the measurement error. This interpretation has also been successfully used in research into physical activity, looking at both the motives for undertaking PA and its effects on physiological variables [18]. Assuming the need to prevent elevated blood pressure and increase physical efficiency in children and adolescents, studies on the variability of responses to physical effort in general and, in particular, the responsiveness of HIIT on blood pressure and cardiorespiratory fitness are a must. From this point of view, searching the cutoff points for effects (meaningful changes) becomes one of the most important components of the field of responsiveness to physical exercises.

Therefore, the purpose of the current work was to determine the MDC of resting blood pressure and cardiorespiratory fitness. Specifically, the aim was threefold: (1) to determine the MDC for SBP, DBP, and FI; (2) to compare the MDC for all measured outcomes; and (3) to determine whether HIIT-induced changes in the analyzed outcomes exceeded the estimated MDC. It was hypothesized that the magnitude of change and individual variability would vary depending on specific variables, which would be reflected in the calculated MDC for each outcome.

## 2. Materials and Methods

### 2.1. Participants

This preplanned secondary data analysis used a subset of data from a two-group experimental design study project called “Physical activity and nutritional education in preventing civilization diseases—theoretical aspects and practical implications for the secondary school physical education program”. It was carried out at a secondary school in Wroclaw, Poland. Recruitment was held at the beginning of September 2018, and the HIIT program was conducted for 10 weeks through the end of September, all of October and November, and completed in December. Measurements were made in the week before and just after the HIIT program. The inclusion criteria were as follows: (a) same age (1st class attendance), (b) no medical or parental requests to not attend PE classes, and (c) voluntary consent of parents or legal guardians for participating in research. The exclusion criteria were as follows: (a) medical contradiction and (b) extra, after school, and sports training. Detailed information about the participants and procedures, as well as the main statistical results (within-group—pre- and postintervention, and between-group—experimental and control groups), have been presented elsewhere [19,20]. The current work is a secondary analysis to study responses to the exercise problem, which began by examining the prevalence of responders and non-responders to the school-based HIIT intervention [21].

Before running the project, G*Power (version 3.1) was used to calculate the a priori sample size. Considering a mixed-effect analysis of variance (ANOVA) as the primary base analysis, an effect size (ES) of 0.25 (medium effect size), a *p*-value of 0.05, a power of 0.80, four groups, and two measurements, the suggested sample size was 179 participants. Initially, the sample comprised 187 adolescents (66 males aged 16.24 ± 0.34 years and 121 females aged 16.12 ± 0.42 years) from a preselected urban comprehensive secondary school in Wroclaw.

**Figure 1:**
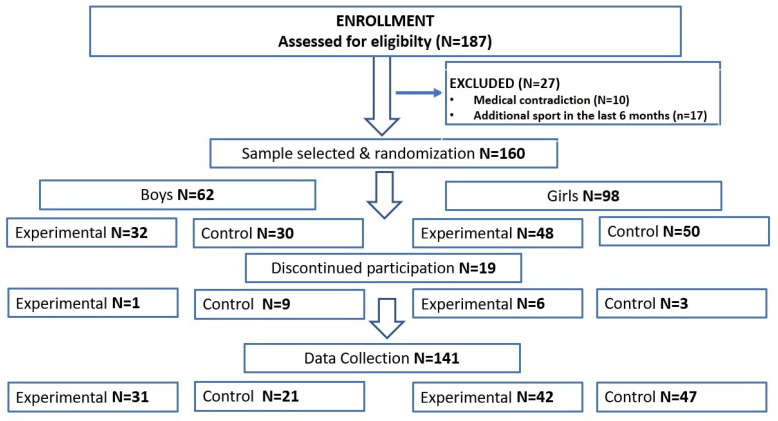
Flowchart diagram: the flow of participants through the study.

The participants comprised six separate classes, of which three were randomly assigned to the experimental group (EG) and three to the control group (CG). Among the 187 participants, 141 participants completed the study, comprising 52 boys (EG N = 31; CG N = 21; age 16.24 (±0.34) years; body height 176.74 (±6.07) cm; body mass 65.42 (±12.51) kg) and 89 girls (EC N = 42; CG N = 47; age 16.12 (±0.42) years; body height 164.38 (±6.54) cm; body mass 56.71 (±10.23) kg). Among the 46 excluded participants, 10 were excluded due to medical contradiction, 17 were excluded from participating in additional sports training, and 19 were excluded during the intervention due to absence in physical education classes. The flow of the participants through the study is presented in Figure 1.

### 2.2. Procedures and HIIT Intervention

The measurements were taken before and after the 10-week intervention on one day from 8:00 a.m. to 1:00 p.m. The intervention lasted 10 weeks. Participants followed the HIIT intervention during one PE lesson (45 min) per week. The HIIT intervention was performed and lasted 14 min, which was divided into three sessions based on the Tabata protocol (20 s work/10 s rest) and separated by a 1 min break. In the first session, participants performed pushups and high knees; in the second session, they performed dynamic lunges and spider crawling; and in the third session, they performed plank-to-pushups and side squeezes. The control group participated in a standard physical education program.

Each participant’s heart rate was measured using a Polar H1 (Polar Electro, Kempele, Finland), and a range of 75–80% HRmax (145–157 heartbeats/min) was established when performing HIIT. The Tanaka formula, HRmax = 208 – 0.7 × “age” (age = 16 years in this study), was used to verify the intensity of the workout. The participants achieved an HR of 156.2 ± 17.8 bpm (CI 95%: 123.0–184.0).

### 2.3. Measurements

#### 2.3.1. Anthropometrical Measurements

Body height (BH) was measured with an accuracy of 0.1 cm using anthropometers (GPM Anthropological Instruments, DKSH Ltd., Zurich, Switzerland). Body weight (BW) was measured using an InBody230 body composition analyzer (InBody Co. Ltd., Cerritos, CA, USA). Based on BH and BW, the body mass index (BMI) was calculated using the following formula: BW[kg]/BH[m]^2^. 

#### 2.3.2. Resting Blood Pressure Measurements

Blood pressure (BP) was measured using an Omron BP710 automatic blood pressure monitor (Omron Healthcare, Inc., Hoffman Estates, IL, USA). The participants had to sit quietly for 10 min. Next, the measurements were taken three times, separated into 10 min intervals. The analyzed results are the means of the three measurements. Systolic (SBP) and diastolic (DBP) blood pressures were recorded.

#### 2.3.3. Fitness Index (FI) (Harvard Step Test)—Cardiorespiratory Fitness (CRF)

The Harvard Step Test (HST) was used to evaluate aerobic capacity. The HST results of the participants were used for the calculation of the fitness index (FI) according to the following formula [22]: FI = (100 × L)/(5.5 × *p*), where L = duration of the test in seconds, L < 300 s, and *p* = heart rate within 1.5 min after the participants stopped the test. The reliability of the HST was acceptable with an intraclass correlation coefficient (ICC) of 0.63 [23]. 

### 2.4. Minimal Detectable Change

The minimal detectable change (MDC) for each of the five variables (BF, BFP, SBP, DBP, and FI) was calculated using the pre–post data of the control group. MDC is defined as the smallest amount of change that is detectable and not due to inherent variation or noise in the measure itself [24,25]. To compute MDC, data from two repeated tests were used. The 90% confidence level of a reliable difference (MDC_90_) is regarded as sufficient for decisions pertaining to the efficacy of clinical interventions [26]. 

To calculate MDC_90_, the intraclass correlation coefficient—ICC(3,1)—and the standard error of measurement (SEM) were calculated first. The following formula for MDC_90_ was then used [25]:

$$\mathrm{SEM}=SD_{\mathrm{baseline}}\sqrt{1-\mathrm{ICC}}$$

$${\mathrm{MDC}}_{90}=1.64\times \mathrm{SEM}\times \sqrt{2}$$

where SEM is the standard error of measurement, SD_baseline_ is the standard deviation of the baseline results, ICC is the intraclass correlation coefficient, and MDC_90_ is the 90%CI of minimal detectable change. 

MDC_90_ was determined to be the appropriate measure of reliability for intervention studies [26]. MDC can be expressed as a percentage (MDC%), an estimate independent of the measurement unit. Representing the relative amount of the random error of measurement, the percentage of the MDC was calculated as (MDC/mean value of each variable) × 100.

### 2.5. Statistics

Data are presented as mean, SD, and 95% CIs. Delta values (Δ) were calculated by subtracting preintervention values from postintervention values. In addition, the percentages of delta (%Δ) were calculated as follows: %Δ = (postintervention–preintervention/preintervention) × 100. The relative values of the changes were used for the purpose of comparing the average differences between the variables. Comparisons between groups (EG vs. CG) were conducted using an independent Student’s *t*-test. Cohen’s *d* was also calculated to assess the effects size of the differences between EG and CG.

The changes induced by HIIT were compared to the calculated MDC values. 

The significance level was set at α = 0.05. The Statistica v. 13.3 statistical package (Tibco, 2023, Cracow, Poland) was used to analyze the study data.

## 3. Results

The pre–post data of 68 participants (31% of boys) in the control group (CG) were used for this preplanned secondary study, and the data from 73 participants (42% of boys) in the experimental group (EG) were used to study the minimal detectable change resulting from the HIIT intervention. In the current work, MDC was studied for the whole groups of participants, regardless of sex. In addition, analysis of the results focused only on changes. Detailed results (mean values and 95%CIs with standard deviations of the pre- and post-intervention measurements) and comparisons considering sexes separately have been published previously [19,20,21]. 

There were no statistically significant differences between the EG and CG (regardless of sex) in the baseline values of body height (EG: 169.81 ± 8.39; CG: 168.00 ± 9.04), body weight (EG: 59.35 ± 10.31; CG: 59.87 ± 12.38), and body mass index (EG: 20.50 ± 2.55; CG: 21.13 ± 3.57) (all *p* > 0.05). Furthermore, no statistically significant differences between groups were observed in changes after the intervention. The participants in both groups were taller after 10 weeks (approximately 2 mm) and heavier (EG = 0.100 kg, CG = 0.470 kg), while BMI slightly decreased in the EG (−0.031) and slightly increased in the CG (0.037) (the *p*-values of the differences were 0.570, 0.177, and 0.480, respectively).

Changes in SBP, DBP and FI are presented as deltas (Δ) and percentages of deltas (%Δ) in Table 1. In the EG, SBP decreased significantly during the HIIT intervention in comparison to the CG. The average change was more than six times higher (Δ = −6.31 vs. Δ = 0.75). This difference was highly statistically significant (*p* < 0.001), and the effect size was very large (Cohen’s d = −1.02). However, the effect of the HIIT was not so obvious in the case of DBP. The difference in the changes in DBP between the EG and CG (Δ = −2.36 vs. Δ = 0.79) was not statistically significant (*p* = 0.107), with a small effect size (Cohen’s d = −0.27). Conversely, the difference in FI between the EG and CG (Δ = 2.36 vs. Δ = 0.35) was again statistically significant (*p* = 0.002), with a moderate effect size (Cohen’s d = 0.54) (Table 1). 

A comparison of the relative changes (%Δ) between the outcomes in the EG after the intervention suggested the highest gain was in FI (5.80%), followed by SBP (−4.88%), with the lowest in DBP (−2.60%). The changes in the CG at the same time were 1.06%, 0.79%, and 0.33%, respectively (Table 1). 

The MDC_90_, MDC_90_%, baseline values (CG), ICC, and SEM are presented in Table 2. The calculated MDC_90_ values for SBP, DBP, and FI were 7.82 and 12.45 mm HG for blood pressure and 5.39 points for FI. The percentages of MDC_90_ for SBP, DBP, and FI were 6.68%, 17.27%, and 12.15%, respectively (Table 2). In general, the relative (%) MDC_90_ values were higher for DBP, while SBP had the lowest values and the values for FI were between the two blood pressure parameters. Therefore, the results clearly showed that the changes in both blood pressure measurements were quite different. This means that there were different reactions to the physical exercise stimulus. 

**Figure 2:**
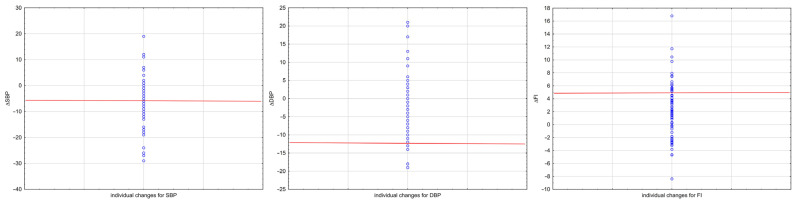
Magnitude of change in SBP, DBP, and FI after HIIT intervention for individuals from experimental group (n = 73). Individual change for each participant is plotted as a function of the MDC_90_ threshold (red horizontal line). Abbreviations: ΔSBP—changes in systolic blood pressure, ΔDBP—changes in diastolic blood pressure, ΔFI—changes in fitness index, MDC_90_—minimal detectable change at 90% of confidence.

For the comparison of HIIT-induced changes and MDC_90_ (presenting a minimal reduction in SBP and DBP and a minimal increase in FI), it was found that each of the three measurements, considering the average values, exceeded its MDC_90_ (Table 2). However, there were some subgroups of participants who individually exceeded the minimal reduction in SBP or DBP or the minimal increase in FI (Figure 2). There were 34.25% of participants who exceeded the MDC_90_ for SBP, while only 5.48% of participants exceeded the minimal detectable change for DBP. A number of participants (21.92%) had a positive change that exceeded the MDC_90_ for FI. This finding confirmed that there was inconsistency regarding the difference in measurement variability within the cardiovascular parameters and a greater similarity between SBP and FI in comparison to DBP.

The mean change for the 25 participants (34.25%) who exceeded the MDC_90_ of SBP was −15.28 ±6.06 mm HG, while the mean change for the 48 participants (65.75%) who did not exceed the MDC_90_ was −1.65 ±5.33 mm Hg. This large difference was statistically significant (*p* < 0.001). Similarly, the mean change for the 4 participants (5.48%) who exceeded the MDC_90_ for DBP was −16.00 ±2.94, while the mean change for the 69 participants (95.52%) who did not exceed the MDC_90_ was −1.57 ±7.95 mm Hg. This difference was also statistically significant (*p* = 0.001). Finally, the mean change for the 16 participants (21.92%) who exceeded the MDC_90_ for FI was 7.76 ±3.09, while the mean change for the 57 participants (78.08%) who did not exceed the MDC_90_ of FI was 0.84 ±2.94. This difference was significant with a *p*-value < 0.001. These findings confirmed the large intra-individual variability among participants in reaction to the intensive physical effort.

## 4. Discussion

The aims of the current study were threefold: (1) to determine the MDC for SBP, DBP, and FI; (2) to compare the MDC for all measured outcomes; and (3) to determine if HIIT-induced changes in the analyzed outcomes exceeded the estimated MDC. To the best of the author’s knowledge, this is the first study to explore meaningful changes in cardiovascular and cardiorespiratory fitness parameters. Another way of analyzing the effect of the HIIT intervention (regardless of sex) confirmed the effectiveness of the school-based HIIT intervention. Data from a previous study were used to compute MDC_90_. The findings showed that the minimal reduction in SBP and DBP was quite different. The increase in FI was similar to the amount of positive change in SBP. Once again, large intra-individual variability was found. This resulted in the conclusion that participants who were more responsive to the exercise stimulus exceeded the MDC_90_ and were significantly different from participants who were not as responsive and did not exceed the MCD_90_.

The benefits of HIIT are usually observed in many health-related fitness outcomes from each of its component: body composition, muscular strength and endurance, and cardiorespiratory fitness [27] are related to outcomes that are often achieved in a few areas; however, there is a lack of deeper insight into individual responses, which may be different due to physiological processes [28,29,30]. 

In the current study, the impact of HIIT was observed in systolic blood pressure and cardiorespiratory fitness, but not in diastolic blood pressure. Similar results were observed by Engel et al. [31], who reviewed four studies involving 577 athletes of similar ages (15.5 ± 2.2 years) who performed HIIT and showed improvements in certain aerobic and anaerobic performance variables. Comparing similar participants and a similar protocol, a study by Racil et al. [32] that examined adolescent females aged 14 revealed improvements in maximal oxygen uptake and maximal aerobic speed following 12 weeks of HIIT or moderate-intensity interval training programs. The same results were also observed in younger (11-year-old) groups [7]. Their protocol was very similar, with HIIT introduced into PE lessons; however, the group was smaller (n = 34). The positive effect of the HIIT program was a reduction in systolic blood pressure. This confirms that high-intensity interval training is a promising tool for improving adolescents’ health [33]. The strength of this method is also linked with a phenomenon related to excess post-exercise oxygen consumption (EPOC), which demands an increased commitment of the cardiorespiratory system and has a greater impact on it, while developing the heart, blood vessels, and lung functions [34,35]. This is the reason why HIIT effort significantly affects metabolism, even a few hours after finishing the session [32,33]. The studies mentioned above are consistent with the current study’s observation of a reduction in systolic blood pressure, but not a reduction in the same amount in diastolic blood pressure. However, in another study, an improvement in both blood pressure parameters was observed [36,37]. In contrast to this study, in which participants conducted one session of HIIT per week, participants conducted 2–3 sessions per week in the aforementioned studies. Therefore, an explanation for the differences in the results may be related to the fact that the HIIT intervention’s effect size depends on training session frequency [38].

The current study again showed large variability in the effects of HIIT on physiological outcomes. The result showing the large differences in response to the exercise stimulus partitions the participants into responders and non-responders to the training. Under the same stimulus, some individuals may achieve different effects, which is related to inter-individual variability in response to exercise training (IVRET) [39,40]. Individual response—a participant’s reaction to the exercise stimulus, which is usually calculated as pre–post difference—is specified as a subject-by-training interaction. However, random measurement error causes background noise, which interferes with the interpretation of differences and has consequences (e.g., in the classification of the response categories) [41]. In the current work, intra-individual variability was assessed by establishing participants who exceeded or did not exceed the MDC_90_ thresholds. First, each outcome showed large variability; second, the variability between the outcomes was different. Similarly, Juric et al. [42] observed a high variability in CRF after an HIIT intervention. Their protocol was very similar, using a cluster randomized control trial design, but it had more participants (n = 207) and assessed a wider range of outcomes. On the other hand, Montero and Lundby [43] showed that participants who did not react should receive a higher load of intervention. The current study showed that the improvement in SBP was similar to the improvement in CRF. This observation is in agreement with Lan et al. [44] and Guo et al. [45], who conducted studies with similar protocols but used different methodologies for studying cardiorespiratory fitness based on exercise tests with maximal voluntary exertion using a treadmill. In addition, the assessment of CRF was based on VO_2max_. 

The methodological approach of studying MDC_90_ for BP and CRF using fitness indexes is unusual. It is not easy to directly compare the results. The calculated MDC_90_ for all outcomes turned out to be high. As a result, the average changes in the experimental group did not exceed the values of MDC_90_. Perhaps this is due to the fact that a large group of participants did not fully respond to the exercise stimulus, e.g., being unable to get fully involved in the training. The ability to withstand external loads is essential, not only in sports but also in daily activities, playing a crucial role in routine life tasks and sports performance [46]. On the other hand, the values of MDC_90_ could have been elevated due to the small number of participants. Yong et al. [47] showed that the MDC_90_ for SBP with a study sample size of 100 was 12 mm HG, while for a sample size of approximately 500 per group, the required MDC_90_ was less than 5 mm HG. The same authors obtained an ICC for BP ranging from 0.84 to 0.94, which is similar to the ICC calculated in this study for SBP (0.86) but not for DBP (0.50). Some authors suggest using pulse pressure instead of raw diastolic blood pressure. The difference in estimated SBP and DBP using noninvasive blood pressure measurements is well known [48]. In the case of CRF, the excellent test–retest reliability has been well documented for the field method measuring VO_2max_ or VO_2peak_ [49,50]. However, the Harvard step test’s reliability is not very good. The correlation with direct VO_2max_ ranged from 0.65 to 0.8 (depending on studies), and reliability was poor (ICC < 0.6) [23]. This observation is in agreement with the current results, in which the ICC for FI was 0.62. Some studies have suggested a minimal detectable change of approximately 204 mL/min (in the case of VO_2peak_) or less than 5% [51,52]. However, these results were obtained from studies with adult men (over 50 years of age). In contrast, the current study showed an MDC_90_ of 12% for FI. In this case, the participants were a mixed group of males and females aged 16 years old.

A strength of this study is that the school-based intervention was implemented in physical education lessons. The findings showed the effectiveness of HIIT introduced in typical lessons in decreasing blood pressure and increasing cardiorespiratory fitness. This work has practical implications for the practice of physical education, showing that this intervention is a proven HIIT program that teachers can freely use. On the other hand, for scientists, the theoretical aspects of such programs require data for comparisons and a foundation for modifying the training programs to increase their effectiveness. Particular attention should be paid to exploring the problem of dose–response effects in terms of SBP and DBP, which react differently to the same stimulus. The potential reason might be related to differences in physiological reactions to physical exercise, thereby showing differences in adaptation. Systolic blood pressure increases linearly with increases in exercise intensity, whereas with most types of training, there is minimal change in diastolic blood pressure. Hence, adaptability, seen as a decrease in resting blood pressure, is noticed in SPB rather than DBP. 

This work also has a few limitations. The small number of participants forced both sexes to be gathered into one group. Future studies should separate the sexes to assess sex-specific minimal detectable changes. The second limitation is using the FI results for cardiorespiratory fitness assessment instead of more precise physiological measurements. The VO_2max_ obtained from well-known field tests (e.g., the beep test) would be more suitable. Even field tests assessing VO_2max_ are more reliable and repeatable than HST. In addition, VO_2max_ is easier to compare between different studies. The next limitation is related to be the largely homogenous ages of the participants. More age categories, particularly adolescents in the prepuberty and puberty periods, would enrich the inference. 

The current work also points to potential new directions that may be worth further exploring. They include exploring gender-specific minimal detectable changes and more diverse age categories. A separate problem could be socioeconomic factors affecting individual variability in physiological measurements. Outcomes could be extended by assessing body mass composition and biochemical measurements. In addition, more advanced measurement methods are required.

## 5. Conclusions

The determination of measurement variability using MDC is useful for assessing meaningful intervention-induced changes. The measurements used in this work are valuable health-related indicators. Still, a growing interest in using school-based programs to fight health problems (excessive body fat, elevated blood pressure, or decreased physical efficiency) in children and adolescents needs verified knowledge about the potential effects of such exercise programs. This is related to identifying responders and non-responders to the intervention as well as minimal detectable change, which can be considered the actual effect of the exercise. Due to the widespread reluctance of young people to exercise, the avoidance of physical effort requires precise definition and proper selection of loads (adjusted to the needs of young people, while not discouraging them from exercising) so that they are not too large and do not last too long but give noticeable effects. In addition, future studies should take into account various additional confounding variables and factors.

## Figures and Tables

**Table 1:** Mean, SD, and 95% (CIs) of changes (Δ) in the experimental group (EG) and control group (CG). The second row contains relative changes (percentages). The results from the *t*-test for independent groups are presented as *t*-values and *p*-values, and Cohen’s values are given at the end (with 95%CI given in the brackets).

Variable	EG				CG				*t*	*p*	Cohen’s d
	Mean ± SD		95%CI		Mean ± SD		95%CI				
ΔSBP	−6.32	−8.31	−4.32	8.56	0.75	−0.38	1.88	4.67	−6.023	<0.001	−1.02 (−1.36, −0.66)
%Δ	−4.88	−6.43	−3.33	6.64	0.79	−0.19	1.76	4.01	−6.076	<0.001	−1.02 (−1.37, −0.67)
ΔDBP	−2.36	−4.32	−0.39	8.43	−0.16	−2.00	1.67	7.59	−1.621	0.107	−0.27 (−0.60, 0.06)
%Δ	−2.60	−5.41	0.21	12.05	0.33	−2.33	3.00	11.01	−1.503	0.135	−0.25 (−0.58, 0.08)
ΔFI	2.36	1.40	3.32	4.13	0.35	−0.42	1.12	3.19	3.223	0.002	0.54 (0.21, 0.88)
%Δ	5.80	3.58	8.02	9.52	1.06	−0.70	2.82	7.27	3.301	0.001	0.56 (0.22, 0.89)

Footnote: %Δ—percentage of change (delta), ΔSBP—change (delta) in systolic blood pressure, ΔDBP—change (delta) in diastolic blood pressure, ΔFI—change (delta) in fitness index, EG—experimental group, CG—control group, SD—standard deviation, CI—confidence interval.

**Table 2:** MDC_90_, baseline values, ICC, and SEM for SBP, DBP, and FI (based on CG results, n = 68).

Variable	Minimal Detectable Change		Baseline				ICC(3,1)	SEM
	MDC_90_	MDC_90_%	Mean ± SD		95%CI			
SBP	7.82	6.68	117.03	9.06	114.83	119.22	0.86 (0.78–0.91)	3.37
DBP	12.45	17.27	72.13	7.58	70.30	73.97	0.50 (0.30–0.66)	5.37
FI	5.39	12.15	44.35	3.77	43.44	45.26	0.62 (0.45–0.75)	2.32

Footnote: SBP—systolic blood pressure, DBP—diastolic blood pressure, FI –fitness index, MDC—minimal detectable change, ICC—intraclass correlation coefficient, SEM—standard error of measurement, CG—control group, SD—standard deviation, CI—confidence interval.

## Data Availability

The data presented in this study are available from the author upon request.
